# Unsupervised Clustering of Individuals Sharing Selective Attentional Focus Using Physiological Synchrony

**DOI:** 10.3389/fnrgo.2021.750248

**Published:** 2022-01-04

**Authors:** Ivo V. Stuldreher, Alexandre Merasli, Nattapong Thammasan, Jan B. F. van Erp, Anne-Marie Brouwer

**Affiliations:** ^1^TNO Human Factors, Netherlands Organisation for Applied Scientific Research (TNO), Soesterberg, Netherlands; ^2^Human Media Interaction, Faculty of Electrical Engineering, Mathematics and Computer Science, University of Twente, Enschede, Netherlands

**Keywords:** unsupervised clustering, unsupervised learning, physiological synchrony, EEG, electrodermal activity, heart rate, inter-subject correlation

## Abstract

Research on brain signals as indicators of a certain attentional state is moving from laboratory environments to everyday settings. Uncovering the attentional focus of individuals in such settings is challenging because there is usually limited information about real-world events, as well as a lack of data from the real-world context at hand that is correctly labeled with respect to individuals' attentional state. In most approaches, such data is needed to train attention monitoring models. We here investigate whether unsupervised clustering can be combined with physiological synchrony in the electroencephalogram (EEG), electrodermal activity (EDA), and heart rate to automatically identify groups of individuals sharing attentional focus without using knowledge of the sensory stimuli or attentional focus of any of the individuals. We used data from an experiment in which 26 participants listened to an audiobook interspersed with emotional sounds and beeps. Thirteen participants were instructed to focus on the narrative of the audiobook and 13 participants were instructed to focus on the interspersed emotional sounds and beeps. We used a broad range of commonly applied dimensionality reduction ordination techniques—further referred to as mappings—in combination with unsupervised clustering algorithms to identify the two groups of individuals sharing attentional focus based on physiological synchrony. Analyses were performed using the three modalities EEG, EDA, and heart rate separately, and using all possible combinations of these modalities. The best unimodal results were obtained when applying clustering algorithms on physiological synchrony data in EEG, yielding a maximum clustering accuracy of 85%. Even though the use of EDA or heart rate by itself did not lead to accuracies significantly higher than chance level, combining EEG with these measures in a multimodal approach generally resulted in higher classification accuracies than when using only EEG. Additionally, classification results of multimodal data were found to be more consistent across algorithms than unimodal data, making algorithm choice less important. Our finding that unsupervised classification into attentional groups is possible is important to support studies on attentional engagement in everyday settings.

## Introduction

Research on brain signals as indicators of mental state, such as attention, is moving from laboratory environments to everyday settings. This comes with several challenges. Firstly, contextual information about the environment and the people acting in it is limited. It is, for instance, usually unknown what events occur in the environment that are of potential interest to individuals. This complicates the process of uncovering attentional state by referring to known events through traditional analysis of event-related brain potentials. Secondly, everyday settings make it difficult to acquire suitable data to train algorithms that uncover mental state. Machine learning techniques have increased our ability to uncover complex mental states even with limited contextual information, but user-specific data from a similar context is required to train well-performing machine learning models. In a supervised machine learning approach, a model is trained with data recorded when information was available about events, and about the mental state of the individuals, to enable discrimination between the mental states of interest for unseen data collected when contextual information is limited. Such paradigms have been widely applied, for instance to recognize the emotional response to videos (Soleymani et al., 2011, 2015), to distinguish between different mental workload conditions (Hogervorst et al., 2014) or to estimate the attentional state of individuals (Abiri et al., 2019; Vortmann et al., 2019). The requirement of context-specific training for discrimination between mental states is the major drawback of supervised machine learning (Aricò et al., 2018). Especially in everyday settings, the ground truth mental state information needed in the training phase is often not available (Brouwer et al., 2015).

We here focus on further exploring an alternative approach that requires little information about the individuals' environment and does not require training. This approach is based on the interdependence of physiological signals in groups of individuals and may be used to probe attentional engagement. A number of everyday settings exist in which groups of individuals share their attention to some degree. An example is a group of students listening to the instruction of a teacher in a classroom. The degree to which physiological signals in such groups of individuals uniformly change is often referred to as physiological synchrony (Palumbo et al., 2017). It has been related to the attentional engagement of individuals in a group, for example when presented with the same narrative stimulus, such as a movie or audio clip (Hasson et al., 2010; Dmochowski et al., 2012). Ki et al. (2016) found that when a participant was actively attending to a movie, his or her electroencephalogram (EEG) was more synchronized with the EEG of others attending to the same movie than when the participant's attention was focused inwardly on a mental arithmetic task. Perez et al. (2020) found similar results when using heart rate instead of EEG. Stuldreher et al. (2020a) found that physiological synchrony in EEG, heart rate and electrodermal activity (EDA) could not only distinguish between different attentional conditions within an individual, but could also distinguish between participants who had received different selective attentional instructions toward the exact same external stimulus. That is, a majority of participants showed more physiological synchrony with others who received the same attentional instructions than with others who received opposite attentional instructions.

Previous work indicates that both similarities in emotional and cognitive processing may underlie physiological synchrony across individuals. Poulsen et al. (2017) found that moments in time with high physiological synchrony often coincided with emotionally arousing scenes of presented movie clips, suggesting that emotional engagement underlies high physiological synchrony. Stuldreher et al. (2020b) showed that not only presentation of emotionally arousing sounds led to high physiological synchrony, but also the presentation of to-be-counted beeps, suggesting shared cognitive processing can also underlie high physiological synchrony. Dmochowski et al. (2014) showed that physiological synchrony over time was predictive of the number of tweets and viewership during a popular television series, where emotional and/or cognitive engagement may have resulted in being compelled to view the stimulus. The contribution of shared emotional or cognitive processing of specific stimuli to the overall interpersonal physiological synchrony seems to depend on the specific physiological measure. Stuldreher et al. (2020b) found that moments of high physiological synchrony in EEG corresponded with the occurrence of cognitive processing, but not with emotionally arousing events. Moments of high physiological synchrony in heart rate, on the other hand, corresponded well with emotionally arousing events, but not with cognitive processing. Nonetheless, physiological synchrony in all of the above measures was shown to distinguish between groups with different selective attentional focus toward the same narrative stimulus (Stuldreher et al., 2020a).

Physiological synchrony thus enables monitoring the degree of attentional engagement without training of a model, and without detailed information about the environment. However, researchers up to now have only identified the specific attentional focus of an individual by putting physiological synchrony in context of other individuals of whom the attentional focus is known, such as inwardly vs. outwardly focused attention (Cohen and Parra, 2016; Ki et al., 2016; Perez et al., 2020) or one of two specific types of selective attentional instructions (Stuldreher et al., 2020a). In everyday settings, such knowledge is not always available. For example, it is not known a priori who out of a group of students are attending to key elements of the lecture, and who are attending to what is happening in the classroom around them, which would be required to classify an unknown individual into one or the other attentional group following the earlier used methods. For such cases, we require unsupervised identification of groups of individuals sharing attentional focus.

Unsupervised learning techniques may be used to find clusters of individuals sharing attentional state. Unlike supervised learning, unsupervised learning techniques are not based on a model that is trained on a labeled dataset. Instead, these techniques form clusters of samples that are proximate in a high-dimensional space (Grira et al., 2004). Numerous algorithms are available, from well-known algorithms such as traditional *k*-means (Lloyd, 1982), and its more modern iterations (Yu et al., 2018; Sinaga and Yang, 2020), to spectral clustering (Von Luxburg, 2007) or hierarchical clustering (Ward, 1963). Complementary to data clustering are ordination techniques, that pre-order objects in such a way so that similar objects are close to each other and dissimilar objects are far away from each other. Often used are the algorithms that are part of the family of multidimensional scaling (Borg et al., 2018).

Unsupervised learning techniques have been explored before in research using physiological measures to assess mental state. For instance, Schultze-Kraft et al. (2016) successfully employed unsupervised learning techniques to classify either low or high operator workload in a laboratory setting based on EEG signals. Existing work focuses on within-subject classification of mental state (Carreiras et al., 2016; Schultze-Kraft et al., 2016; Maaoui and Pruski, 2018). To the best of our knowledge, unsupervised clustering of individuals sharing their attentional focus has not been demonstrated before.

The goals of the current work are therefore two-fold. First, we establish the feasibility of unsupervised clustering of individuals based on physiological synchrony, to automatically identify groups of individuals sharing attentional focus without pre-knowledge of attentional focus of any of the individuals. Clustering performance is evaluated by using ground truth information on attentional state. Second, we investigate how performance depends on the type of physiological measure used. While distinguishing between different attentional conditions using synchrony in EEG, EDA, and heart rate has been explored before (Stuldreher et al., 2020a), we do not know how such results transfer to an unsupervised approach. Additionally, we test performance when multiple physiological measures are combined. We investigate all of this with the use of a broad range of classic and more modern unsupervised learning techniques. A secondary goal therefore is to compare clustering performance across algorithms.

In this study, we use the (Stuldreher et al., 2020a) publicly available dataset (https://osf.io/8kh36/) in which ground-truth information about the attentional state of individuals is available. Though such information is generally not available in everyday—and if it is, one would use supervised learning techniques due to their higher performance compared to unsupervised learning (Blankertz et al., 2016; Aricò et al., 2018)—we here need the ground truth information to reflect on the performance of this novel approach. We also investigate the use of the silhouette coefficient as a potential way to evaluate unsupervised clustering performance in scenarios where no ground-truth information is available (Rousseeuw, 1987).

In sum, we investigate whether attentional focus can be determined using unsupervised clustering, and if so, whether clustering performance depends on the type of physiological modality (EEG, EDA, and heart rate).

We hypothesize that:

1) Attentional focus can be determined using unsupervised clustering techniques.
2) Classification accuracies are higher when using EEG rather than EDA or heart rate.
3)Combining modalities into a multimodal approach leads to higher classification accuracies than unimodal approaches because a multimodal approach includes information of more mental processes in the classification decision.
4) The silhouette coefficient is correlated with clustering accuracy.

When testing these hypotheses, we use multiple clustering algorithms. An additional exploratory research question is how performance depends on clustering algorithm.

## Methods

### Participants

Twenty-seven participants (17 female), aged between 18 and 48 years (*M* = 31.6, *SD* = 9.8 years), took part in the experiment. They were recruited through the participant pool of the research institute where the study took place. None of the participants reported problems with hearing. Prior to the experiment all participants signed an informed consent, in accordance with the Declaration of Helsinki. All participants received a small monetary compensation for their participation in the experiment and for traveling costs. Data from 26 participants were further processed due to a recording failure in one case. The experiment was approved by the TNO Institutional Review Board. The approval is registered under reference 2018-70.

### Materials

Electroencephalogram, EDA, and electrocardiogram (ECG) were recorded at 1,024 Hz using an ActiveTwo Mk II system (BioSemi, Amsterdam, Netherlands). Electroencephalogram was recorded with 32 active Ag/AgCl electrodes, placed on the scalp according to the 10–20 system, together with a common mode sense active electrode and driven right leg passive electrode for referencing. The electrode impedance was maintained below 20 kOhm. For EDA, two passive gelled Nihon Kohden electrodes were placed on the ventral side of the distal phalanges of the middle and index finger. For ECG, two active gelled Ag/-AgCl electrodes were placed at the right clavicle and lowest floating left rib. Electrodermal activity and heart rate were also recorded using wearable systems (Movisens EdaMove 4 and Wahoo Tickr, respectively). These data are not discussed here, but are publicly available on https://osf.io/8kh36/ and compared to the data recorded using the ActiveTwo in van Beers et al. (2020).

### Stimuli and Design

Participants performed the experiment one by one. Each participant listened to the same audio file, composed of a 66 min audiobook (a Dutch thriller “Zure koekjes,” written by Corine Hartman) interspersed with other auditory stimuli. The 13 participants in the audiobook attending (AA) group were asked to focus on the narrative of the audiobook and ignore all other stimuli or instructions. The 13 participants in the stimulus attending (SA) group were asked to focus on the other stimuli, perform accompanying tasks, and ignore the audiobook. The order of interspersed stimuli was randomly determined, but was identical for each participant. Intervals between the end of one stimulus and the onset of the next one varied between 35 and 55 s (*M* = 45, *SD* = 6.1 s). The short auditory stimuli were affective sounds, blocks of beeps, and the instruction to sing a song. For the exact types and order of interspersed stimuli we refer the reader to Stuldreher et al. (2020a).

After the experiment, all participants were asked to answer two questionnaires. In the first questionnaire, participants used a slider on a horizontal visual analog scale running from “not at all” to “extremely” to rate their mental effort, distraction and emotion during the short emotional sounds. The second questionnaire was on the content of the stimuli: participants were asked to report as many emotional sounds as they could remember, they were asked to estimate the average number of beeps in a block, and they were asked questions about the content of the narrative. For more details we refer the reader to Stuldreher et al. (2020a).

### Analysis

**Figure 1:**
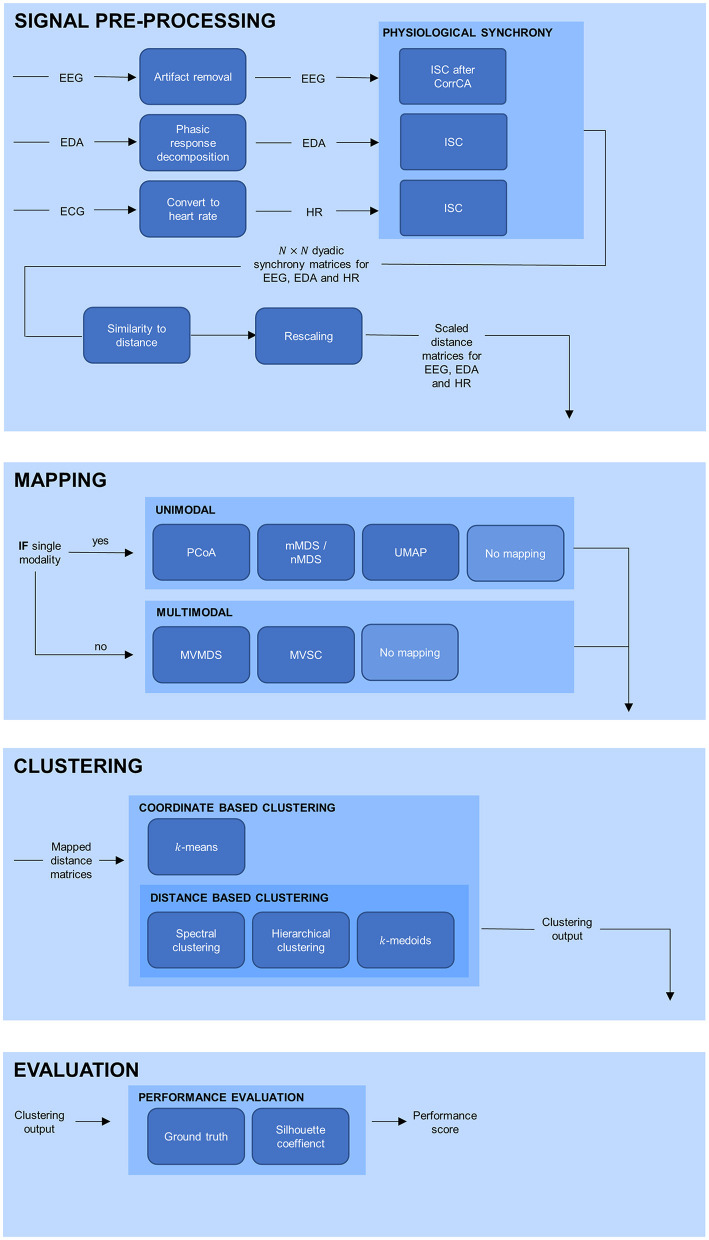
Overview of the processing pipeline, divided in signal pre-processing, mapping, clustering, and evaluation. EEG, electroencephalogram; EDA, electrodermal activity; ECG, electrocardiogram; HR, heart rate; PS, physiological synchrony; PCoA, principle coordinate analysis; mMDS, metric multidimensional scaling; nMDS, non-metric multidimensional scaling; UMAP, uniform manifold approximation and projection; MVMDS, multiview multidimensional scaling; MVSC, multiview spectral clustering.

An outline of the complete analysis is depicted in Figure 1. In the sections below, each part of the analysis is elaborated upon separately.

#### Signal Pre-processing

Data pre-processing was done using MATLAB 2019a software (Mathworks, Natick, MA, USA). Electroencephalogram was pre-processed using EEGLAB v14.1.2 for MATLAB (Delorme and Makeig, 2004). To remove potentials not reflecting sources of neural activity, like ocular or muscle-related artifacts, logistic infomax independent component analysis (Bell and Sejnowski, 1995) was performed. Electroencephalogram was first downsampled to 256 Hz and high-pass filtered with the passband edge at 1 Hz using the standard finite-impulse-response filter implemented in EEGLAB function pop_eegfiltnew. This relatively high cut-off frequency has shown to work better for independent component analysis compared to lower cut-off frequencies (Winkler et al., 2015). Data were then notch filtered at 50 Hz, again using the standard finite-impulse-response filter implemented in EEGLAB function pop_eegfiltnew. Channels were re-referenced to the average channel value. Independent component analysis was performed and the Multiple Artifact Rejection Algorithm (Winkler et al., 2011) was used to classify artifactual independent components. Components that were marked as artifactual were removed from the data. Then, samples whose squared amplitude magnitude exceeded the mean-squared amplitude of that channel by more than four standard deviations were marked as missing data (“NaN”) in an iterative way with four repetitions to remove outliers. By doing so, 0.8% of data were marked as missing.

Electrodermal activity was downsampled to 32 Hz. The fast changing phasic and slowly varying tonic components of the signal were extracted using Continuous Decomposition Analysis as implemented in the Ledalab toolbox for MATLAB (Benedek and Kaernbach, 2010). In further analyses we use the phasic component, as this component of the EDA signal is mainly related to responses to external stimuli (Boucsein, 2012).

Electrocardiogram measurements were processed to acquire the inter-beat interval (inversely proportional to heart rate). After downsampling to 256 Hz, ECG was high-pass filtered at 0.5 Hz. R-peaks of the ECG signal were detected following Pan and Tompkins (1985), resulting in a semi-time series of consecutive inter-beat intervals. This inter-beat interval semi-time series was transformed into a time series by interpolating consecutive intervals at 32 Hz.

#### Physiological Synchrony

We computed inter-subject correlations in the time-domain as a measure of physiological synchrony. Rather than treating EEG signals separately, the inter-subject correlations were evaluated in the correlated components of the EEG (Dmochowski et al., 2012, 2014). The goal of the correlated component analysis is to find underlying neural sources that are maximally correlated between participants, using linear combinations of electrodes. The technique is similar to the more familiar principal component analysis, differing in that projections capture maximal correlation between sets of data instead of maximal variance within a set of data. After computing the correlated components based on data from all 26 participants, EEG data of each participant were projected on the component vectors. Inter-subject correlations between a participant with all other participants were then computed as the sum of correlations in the first three component projections, as correlations in higher order projections are often close to chance level (Ki et al., 2016). The result is a *N* × *N* matrix inter-subject correlations of all possible pairs of participants. The correlation values were normalized by dividing all correlation values by the diagonal value—in this case three, as we computed physiological synchrony as the sum of correlations in the first three correlated components.

For EDA and heart rate, we also computed inter-subject correlations in the time-domain as a measure of physiological synchrony. Pearson correlations were calculated over successive, running 15 s windows at 1 s increments. The overall correlation between two participants was computed as the natural logarithm of the sum of all positive correlations divided by the sum of the absolute values of all negative correlations. Again the correlation matrices were normalized. Originally, the diagonal here contained infinite values (as there are no negative correlations, the denominator in the ratio is zero). We therefore chose to replace these cells with finite values in such a way that the ratio between the diagonal value and the mean of the matrix was the same for the matrices of EDA and heart rate as for EEG. Then again, all correlations were divided by the diagonal value.

Clustering algorithms usually require distance matrices. Thus, correlation matrices were transformed into distance matrices before applying clustering algorithms. Several transformations exist (Groenen and van de Velden, 2004). We followed the suggestion of Gower and Legendre (1986) and computed the values in the distance matrix as the square root of one minus the values in the correlation matrix.

As the off-diagonal correlation values were close to zero, and thus the off-diagonal distance values close to one, we applied a linear transformation of each off-diagonal coefficient like in interval multidimensional scaling (Borg and Groenen, 2005) to evenly distribute the values between zero and one.

#### Mapping

Various ordination methods, or “mappings,” have been proposed to create distance matrices. Mapping is complementary to data clustering in such way that objects are ordered so that similar objects are close to each other and dissimilar objects are far away from each other. Mappings toward a different (lower-dimensional) space can be of value for visualization of clusters, and they can improve clustering performance (Kent et al., 1979). The dimension of the mapped space can be chosen arbitrarily. We chose the output mapping to be in two-dimensional space, which is most common in literature and easy to interpret. We applied different, commonly known mappings, of which an overview can be found in Table 1.

**Table 1:** Overview of the used ordination techniques.

**Method**	**Description**	**Reference**
Principle Coordinate Analysis (PCoA)	Also referred to as classical multidimensional scaling, PCoA intends to preserve the distances in the distance matrix in the output mapping. To do so, for each participant the objective is to find coordinates in a lower dimensional space that minimize the strain with the original values.	Groenen and Borg, 2014
Metric Multidimensional Scaling (mMDS)	mMDS is a superset of the PCoA that generalizes the optimization procedure, where instead of strain often the stress is minimized. The minimization problem is solved iteratively as there exists no exact solution.	Borg and Groenen, 2005
Non-metric Multidimensional Scaling (nMDS)	Unlike PCoA, nMDS distorts the distances in the ordination solution. However, it preserves the rank of dissimilarities by minimizing the non-metric stress in an iterative approach.	Kruskal, 1964
Uniform Manifold Approximation and Projection (UMAP)	• UMAP is a non-linear manifold learning technique originally developed as dimensionality reduction. It emphasizes local distances over global distances. • As the UMAP algorithm is also able to deal with ground-truth information known about some of the data points, it can either be used as all other methods or with self-supervised learning (SSL). With SSL, at the algorithm initialization, no labels are known. When the first mapping and clustering are done, a known label is assigned to the participant which is the closest to one of the cluster center. This procedure is then repeated, each time adding the participant closest to one of the cluster centers that has not been labeled yet.	McInnes et al., 2018
Multiview Multidimensional Scaling (MVMDS)	Multi view dimensionality reduction solutions have emerged to solve problems where various samples of the same observation are collected, as is the case here with synchrony in EEG, EDA, and heart rate. MVMDS is a multimodal extension of PCoA—with only one matrix as input, results are identical—that intends to find the common eigenvectors across the different distance matrices	Trendafilov, 2010
Multiview Spectral Clustering (mvSC).	Like MVMDS, this technique is a multimodal extension of the spectral clustering ordination. It computes the common eigenvectors of the Laplacian of the dissimilarity matrices.	Kanaan-Izquierdo et al., 2018

#### Clustering

After mapping, or skipping the mapping, we applied a range of classical clustering algorithms (Table 2). Not all combinations of mapping and clustering yielded valid results. Some methods, for example, are not deterministic, but provide different outcome maps for different initializations. We therefore used multiple random initializations and averaged over the clustering results for each initialization. However, this approach did not converge when using *k*-means on the raw distance matrices.

**Table 2:** Overview of used clustering algorithms.

**Method**	**Description**	**Reference**
*k*-means	Probably the best known clustering algorithm, *k*-means aims to partition all *n* observations into *k* clusters (here *k* = 2), in which each observation belongs to the cluster with the nearest mean. Solutions are found iteratively.	Lloyd, 1982
*k*-medoids	This adaptation of *k*-means is based on the same principle, but rather than minimizing the distance between data points and the cluster center, that is not necessarily one of the input data points, *k*-medoids chooses data points as centers and minimizes the distance between data points and this medoid.	Bauckhage, 2015
Spectral clustering	This technique makes use of the spectrum—or eigenvalues—of the similarity matrix to perform dimensionality reduction before clustering using traditional algorithms like *k*-means. Therefore, this algorithm somewhat combines a mapping and clustering algorithm in one.	Von Luxburg, 2007
Hierarchical clustering	As the name suggests, this algorithm builds a hierarchy of clusters in a bottom-up fashion. Initially, each data point thus belongs to its own cluster. The clusters are progressively merged according to similarity criteria called linkage. We here use Ward linkage that finds new clusters by minimizing the sum of squared differences within the merged clusters.	Ward, 1963

#### Evaluation: Clustering Quality Assessment

To assess the clustering quality we compared found clusters to attentional condition labels (AA or short SA), so that the clustering performance can easily be assessed. To investigate whether clustering performance is above chance level, we conducted a permutation analysis with shuffled group-labels, so that we can compare the clustering accuracy to accuracies obtained for 100 trials with randomized group-labels. We determined the significance level using a one-tailed non-parametric Mann Whitney U-Test. Chance level distributions were determined for all algorithm combinations. The threshold for significantly higher clustering accuracies compared to chance were found to be either 65% (17 out of 26 participants correctly clustered) or 70% (18 out of 26 participants correctly clustered). We selected the strictest significance level (i.e., 70%) to compare all classification results to.

In real-world conditions, ground-truth information on the attentional state is often not a-priori available, which makes it hard to tell how well-unsupervised clustering of attentional states works in a particular condition. Therefore, we explored an alternative measure of evaluating clustering performance, known as the silhouette coefficient (Rousseeuw, 1987). This index measures the compactness and separation of clusters and may be informative as a confidence metric of the clustering outcome. A confident clustering outcome would be associated with tight and well-separated clusters, depicted by a silhouette coefficient near one, whereas an unconfident clustering outcome would be associated with broadly spread and overlapping clusters, depicted by a silhouette coefficient near zero. The silhouette coefficient cannot be determined for all combinations of mapping and clustering methods, for example when using random initializations before mapping over which has to be averaged, as can be de the case with nMDS, mMDS, or UMAP.

## Results

### Clustering Performance Using Physiological Synchrony in Either EEG, EDA, or Heart Rate

**Figure 2:**
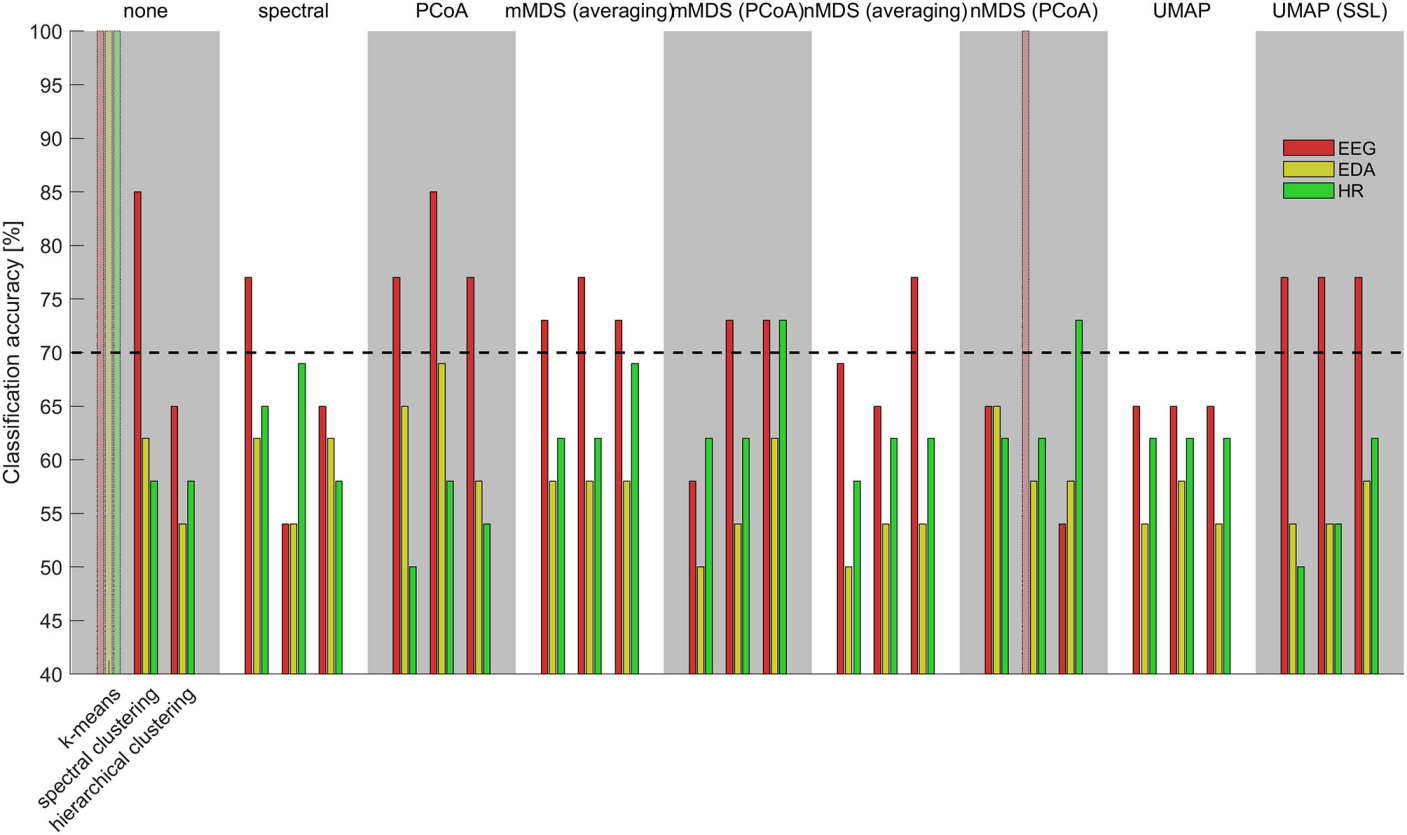
Clustering accuracies utilizing physiological synchrony in EEG (red), EDA (yellow), heart rate (green) for different combinations of mappings (top-axis) and clustering methods (bottom-axis). Transparent top-to-bottom bars represent missing data. The dashed black line depicts significance level compared to chance level classification accuracies.

A complete overview of clustering performance for all used combinations of mapping algorithms and clustering algorithms based on physiological synchrony in either EEG, EDA, or heart rate is presented in Supplementary Table A1. It shows the clustering accuracy, the misclassified participant IDs and silhouette coefficient, wherever available. Figure 2 visualizes the clustering accuracies across the eight mapping methods and the three clustering methods that could all be combined with each other, as well as results when using no mapping, for which we could determine results for two out of the three clustering methods. Classification accuracies above the black line are significantly higher than chance level. That is, classification accuracies of 70% or higher are significantly higher than the chance level distribution at *p* < 0.05. The best performance is obtained using physiological synchrony in EEG [Mdn = 73%, Inter Quartile Range (IQR) = 12% across algorithms], with a maximum clustering accuracy of 85% when using spectral clustering on the raw distance matrix or after applying PCoA ordination. For EDA, a median performance of 58% (IQR = 8%) was obtained; best EDA performance was reached using *k*-means with nMDS mapping (65%). For heart rate, median performance was 62% (IQR = 4%); best performance was reached using hierarchical clustering with nMDS or mMDS mapping (73%).

We determined the silhouette coefficient as a potential alternative measure of clustering performance. The results in Supplementary Table A1 do not suggest that a high silhouette coefficient corresponds with high clustering accuracy as evaluated using knowledge of the attentional instruction groups. This impression is confirmed by a lack of correlation between clustering accuracy and silhouette coefficient (*r* = 0.06, *p* = 0.543). We can note, however, that the silhouette coefficient is generally higher after mapping (Mdn = 0.33, IQR = 0.04) than without mapping (Mdn = 0.13, IQR = 0.13).

### Clustering Performance Combining Physiological Synchrony in EEG, EDA, and Heart Rate

**Figure 3:**
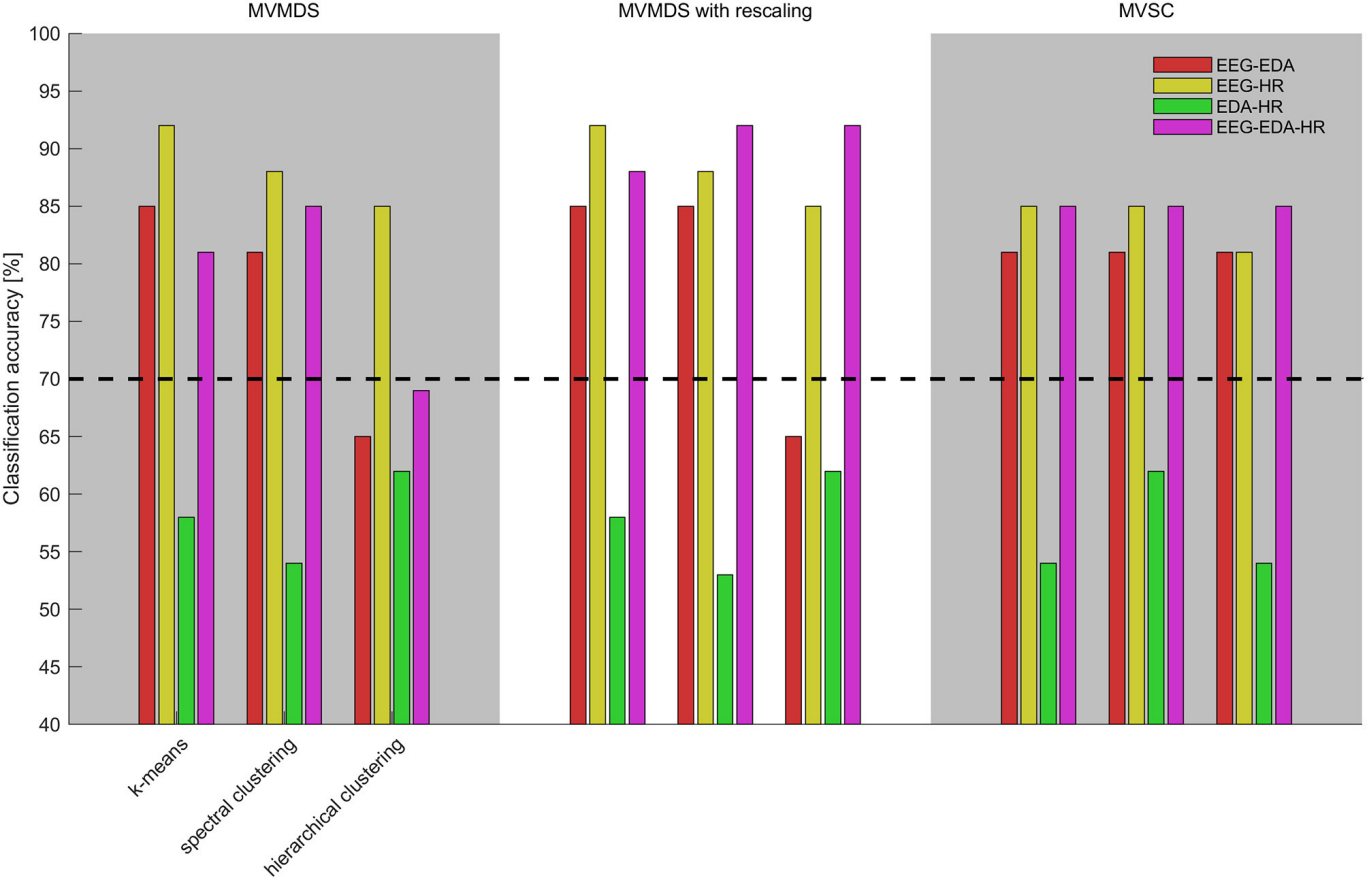
Clustering accuracies utilizing physiological synchrony in EEG and EDA (red), EEG and HR (yellow), EDA and heart rate (green), and EDA, EDA, and heart rate (purple) for different combinations of mappings (top-axis) and clustering algorithms (bottom-axis). The dashed black line depicts significance level compared to chance level classification accuracies.

Supplementary Table A2 presents the clustering results when combining physiological synchrony in multiple modalities for all possible mapping-clustering combinations. It shows clustering accuracies, misclassified participant IDs and silhouette coefficient when combining EEG and EDA, EEG and heart rate, EDA and heart rate, and all three modalities. Figure 3 presents an overview of the accuracies for each mapping-clustering combination. Again, the dashed black line at 70% depicts significance level compared to chance. The best clustering performance of 92% is reached for EEG combined with heart rate when *k*-means and MVMDS or MVMDS-with-rescaling are used; as well as for the combination of EEG, heart rate, and EDA when MVMDS with rescaling is used with spectral or hierarchical clustering.

Table 3 shows statistical comparisons of classification accuracy between single modality (EEG, EDA, or heart rate) to all other multimodal combinations. Adding modalities increases performance, except when EDA is complemented with heart rate, or heart rate with EDA. Combinations of EDA and heart rate results in median clustering accuracy of 58% (IQR = 8%).

**Table 3:** Test statistics of comparison between classification results using different combinations of physiological measures.

**EEG vs. EEG – EDA**	**EEG vs.** **EEG – HR**	**EEG vs.** **EDA – HR**	**EEG vs. EEG – EDA – HR**
*t*(37) = −2.77, *p* = 0.009	*t*(37) = −5.77, *p* < 0.001	*t*(37) = 4.40, *p* = < 0.001	*t*(37) = −4.78, *p* = < 0.001
**EDA vs. EEG – EDA**	**EDA vs. EEG** **– HR**	**EDA vs. EDA** **– HR**	**EDA vs. EEG** **– EDA – HR**
*t*(38) = −10.92, *p* = < 0.001	*t*(38) = −19.08, *p* < 0.001	*t*(38) = −0.22, *p* = 0.825	*t*(38) = −14.91, *p* < 0.001
**HR vs. EEG – EDA**	**HR vs. EEG** **– HR**	**HR vs. EDA** **– HR**	**HR vs. EEG** **– EDA – HR**
*t*(38) = −7.61, *p* < 0.001	*t*(38) = −12.88, *p* < 0.001	*t*(38) = 1.66, *p* = 0.104	*t*(38) = −10.61, *p* < 0.001

*Gray cells depict significant differences*.

While adding other modalities to EEG results in higher clustering performance, perhaps more important is that clustering performance seems more robust across algorithms. When combining EEG with EDA (Mdn = 81%) IQR is 4%; when combining EEG with heart rate (Mdn = 85%), IQR is 3% whereas IQR is 12% when using EEG only. When combining all three metrics, performance is as consistent as when combing EEG with heart rate only (Mdn = 85%, IQR = 3%).

### Comparing Clustering Performance With Other Measures Reflective of Attentional Engagement

Even though we specified the attentional instructions in the current study, we should note that we cannot be sure the attentional focus of participants is always as specified in the instructions. An incorrect classification may therefore not necessarily mean that the algorithms provided the wrong output, it may also be the case that the incorrectly classified participants did not follow their attentional instructions. To explore this possibility, we examined whether participants that were incorrectly classified by the majority of the methods for EEG performed worse on performance measures reflective of their attentional focus (number of correctly answered questions about the content of the narrative, number of correctly described emotional sounds, estimated number of averagely presented beeps), than participants that were correctly classified by the majority of the methods for EEG.

Seven participants were misclassified for more than 50% of the methods and designated as “often misclassified” (ID's: 2, 3, 8, 10, 16, 18, 25). Table 4 provides the performance characteristics of often misclassified and often correctly classified participants and test statistics comparing the two. In the SA group, participants that were often misclassified described significantly less emotional sounds correctly than participants that were often correctly classified, which indeed suggests that misclassified SA instructed participants did not attend to the emotional sounds very well. For the other two performance measures no significant differences were found.

**Table 4:** Test statistics comparing performance on questions reflective of attentional focus of the often incorrectly classified participants and the often correctly classified participants.

	**Often correct** **participants AA**	**Often incorrect** **participants AA**	**Often correct** **participants SA**	**Often incorrect** **participants SA**
Number of correctly answered narrative questions	Mdn = 5, IQR = 3	Mdn = 6.5, IQR = 2.5		
	*W* = −57, *p* = 0.445			
Number of reproduced affective sounds			**Mdn = 7, IQR = 6**	**Mdn = 4, IQR = 2.3**
			**W=83.5, p=.028**	
Difference between average number of estimated beeps and true number of beeps			Mdn = 2.5, IQR = 13	Mdn = 1, IQR = 9
			*W* = −74.5, *p* = 0.475	

*Cells with bold text refer to significant differences. The grey shades correspond to incompatible combinations*.

## Discussion

We here showed that by applying unsupervised learning techniques to physiological synchrony, groups of participants sharing selective attentional focus can be identified from a set of participants with one of two different selective attentional instructions. This confirms hypothesis 1. Obtained results were found to depend on the physiological modality on which clustering was based.

### Clustering Performance Using Physiological Synchrony in Either EEG, EDA, or Heart Rate

We hypothesized that in line with previous research on physiological synchrony, from the three physiological measures EEG would perform best (hypothesis 2). Indeed, with the use of EEG, best performance was obtained. The maximum classification accuracy was 85% which is well above the threshold of 70% above which classification is significantly higher than chance level. However, performance varied strongly across clustering algorithms, with accuracies as low as 54% for some of the algorithms used.

Applying the clustering algorithms to physiological synchrony in EDA or heart rate resulted in lower classification accuracies than in EEG, and generally led to performance near theoretical chance level. This is in line with other work, where synchronous changes in peripheral modalities have been shown to reflect attentional engagement with narrative stimuli less robustly than EEG (Ki et al., 2016; Perez et al., 2020; Stuldreher et al., 2020a; Madsen and Parra, 2021).

### Effect of Multimodal Combination of Physiological Measures on Clustering Performance

We hypothesized that combining modalities in a multimodal approach would enhance clustering performance compared to a unimodal approach, because different modalities capture different underlying mental processes (hypothesis 3). We partly accept this hypothesis. Indeed, when combining heart rate and EEG, EDA and EEG, or heart rate, EDA, and EEG, the clustering accuracy for combined modalities is higher than when using either of the modalities alone (Table 3). When combining heart rate and EEG, or heart rate, EDA, and EEG, the best obtained clustering accuracy across algorithms was also higher than when using either of the measures alone. When combining EDA with heart rate, classification accuracies were not higher compared to EDA or heart rate alone and thus still did not exceed chance level. Importantly, we found that when combining multiple physiological measures, results were not only generally higher but also more consistent across the range of mapping and clustering approaches. This was even the case when combination of modalities did not increase maximum classification accuracies, as for the combination of EEG and EDA. Thus, a multimodal approach resulted in classification performance that is less dependent on the specific algorithm choice. This observation advocates a multimodal approach in everyday settings where for unimodal data, the patterns of variation in algorithm performance may be different than the ones found here.

### Factors Underlying Performance Differences Between Modalities

We found that identifying two attentional groups in our study works best when physiological synchrony in EEG is used rather than EDA and heart rate. As mentioned in the introduction, we previously found that inter-subject correlations in EEG were especially sensitive to well-timed events inducing top-down modulation of attention, more so than to emotional sounds attracting attention bottom-up (Stuldreher et al., 2020b). This and related work showed major pre-frontal and parietal components contributing to inter-subject correlations in EEG when attending to narrative stimuli (Dmochowski et al., 2012; Cohen and Parra, 2016; Ki et al., 2016). Exactly these cortical areas are of major importance in top-down conscious attention processing (Vuilleumier and Driver, 2007). Inter-subject correlations in EDA and heart rate were modulated more by emotional sounds attracting attention bottom-up than by events that caused top-down modulation of attention (Stuldreher et al., 2020b). Other work also suggests that autonomic synchrony during presentation of narrative stimuli is mostly linked with emotional processing of these stimuli (Golland et al., 2014; Steiger et al., 2019). Electrodermal activity and heart rate are largely innervated by midbrain structures, such as the hypothalamus, amygdala and insula (Thayer et al., 2009; Boucsein, 2012) that are hard to capture using EEG. Such midbrain structures have been related strongly to bottom-up emotional modulation of attention (Behrmann et al., 2004; Vuilleumier and Driver, 2007). The fact that in our study, the difference between attentional groups was induced by instructions that affected attention in a cognitive, top-down manner, may have led to the finding that inter-subject correlations in EEG can here better distinguish between the groups with different selective top-down attentional conditions than inter-subject correlations in EDA and heart rate. Future work should investigate whether inter-subject correlations in EDA and heart rate are more suitable than EEG to distinguish between groups with different attentional conditions driven by emotional.

While physiological synchrony in EEG was found to be most informative of attentional group, adding other modalities generally led to higher and more robust performance. We see two potential explanations for the more robust clustering performance when combining modalities. It may be so that combining multiple modalities compensates for potential noisy observations in any of the modalities. Recent work of Madsen and Parra (2021) showed that physiological synchrony in EEG and heart rate in response to instructional videos are co-modulated. Thus, one noisy measurement may be compensated for by another measurement. Alternatively, more robust performance when combining modalities is expected when the physiological measures reflect different aspects of attentional engagement, so that by combining modalities in a multimodal fashion, one captures more aspects of the shared attentional engagement.

### Effect of Mapping and Clustering Approach on Clustering Performance

In our study, best classification results were obtained when using ordination techniques, here referred to as mapping methods, before applying clustering algorithms compared to directly using clustering algorithms on the distance matrices. This observation is supported by the silhouette coefficient, a measure of compactness and separability of the clusters, indicating less separable clusters when directly applying a clustering algorithm on the distance matrices than when using mapping methods. The low separability of clusters in the raw distance matrices may also explain why clustering results obtained with methods like *k*-medoids, that has random initializations, are different for different runs with different initializations and often did not converge. The only algorithm that provides good results when directly applied on the distance matrices is spectral clustering. This supports the notion that mapping before clustering is important, as the spectral clustering algorithm itself already computes a map before applying a clustering algorithm.

We cannot pinpoint the best mapping method for a general case. Here, clustering performance generally was best using PCoA or the multimodal equivalent MVMDS before applying clustering algorithms. Future work would have to show whether these findings are generalizable across use cases and physiological synchrony computation choices.

When using only a single modality, performance is strongly depends on the mapping method, and to a lesser degree on the clustering algorithm. With the exception of spectral mapping and no mapping conditions, clustering accuracy difference between the best and worst performing clustering algorithm with the same mapping is only around 15%. We cannot pinpoint a single clustering algorithm that performs best for each mapping.

### Evaluation of Clustering Performance

In the current study we could employ the known attentional instructions to evaluate clustering performance. However, as noted before, we cannot be sure that the attentional focus always corresponded to the instructions. An incorrect classification may therefore not necessarily mean that the algorithms provided the wrong output, it may also be the case that the incorrectly classified participants did not follow their attentional instructions. We examined whether participants that were incorrectly classified by the majority of the methods for EEG scored worse on performance measures reflective of their instructed attentional focus (number of correctly answered questions about the content of the narrative for NA participants, number of correctly described emotional sounds, and estimated number of averagely presented beeps for SA participants). Indeed, we found that often incorrectly classified SA participants performed worse on the retention of the emotional sounds than the other participants, though there was no difference for the other two measures.

In real-world applications where unsupervised methods as proposed in the current work may be most applicable, the ground truth attentional condition is often not available. We therefore investigated whether the silhouette coefficient, a measure of the separability of the found clusters, may be used as a reliable metric of clustering performance. Unfortunately, a higher classification accuracy did not correspond to a higher silhouette coefficient and vice-versa. We thus reject hypothesis 4.

Since we have no reliable metric to evaluate clustering reliability when ground-truth labels are not known in real-world use cases, we suggest the use of a multimodal approach when applying unsupervised clustering algorithms on physiological synchrony data. Our results show that a multimodal approach is less prone to incorrect results that can occur for specific algorithm choices.

### Future Work

In this study, we sought to identify two clusters as participants were instructed to either attend to the audiobook or to the interspersed stimuli. However, it may be the case that some participants did not attend well to any of the presented information, a proposition that is substantiated by the observation that some participants showed low synchrony with both the audiobook and stimulus-attending groups and answered questions on the content of both presented streams of information well below the average (Stuldreher et al., 2020a). Others may have attended to both the audiobook and the interspersed stimuli. These two types of participants not necessarily fall into one of the two attentional groups considered in the current work, and may have negatively impacted the clustering performance, especially in algorithms such as *k*-means, where classification is strongly influenced by extreme values (Gupta et al., 2017). For more realistic clusters and better applicability in real-world environments, future work should evaluate clustering performance in relation to varying the numbers of clusters. Possible ways to approach this would be to take into account non-attending participants beforehand, by using outlier detection (He et al., 2003), to pre-specify three or four clusters in input of the clustering algorithm (e.g., to take into account non-attending participants and all-attending participants beforehand), or to use algorithms like mean shift (Comaniciu and Meer, 2002) or DBSCAN (Schubert et al., 2017) that automatically determine how many clusters appear in the data.

Another issue not addressed in current work is that many algorithms—such as *k*-means—tend to provide equal-sized clusters. This effect was not damaging in our study because we expected that the true clusters were about equal size, but in cases where this is not the case, results might be influenced by this algorithm bias. Future work should investigate how unequally sized clusters influence results and should explore algorithms that are less prone to such bias.

Future work should also explore other metrics for the assessment of clustering quality. In the current work the silhouette coefficient did not correspond well to ground truth performance. Potential metrics are distance to the cluster centroid or focus on clusters borders.

Finally, from a mathematical point of view, using other ways of computing the synchrony between the physiological signals could help improving clustering performance. In the current work, simple Pearson correlations were used to compute synchrony between two time-series, but computation of meaningful physiological synchrony, and therewith clustering performance, may be enhanced using other methods such as Dynamic time warping (Berndt and Clifford, 1994). Computing the correlation between two high dimensional signals can lead to the curse of dimensionality, a phenomenon that occurs in clustering with high-dimensional data, where data are more uniformly spread in high dimensions compared to lower dimensions when using a classical distance measure such as Euclidean distance (Bellman, 1966). Dynamic time warping was constructed with the aim of avoiding the curse of dimensionality, which could potentially lead to better clustering results.

## Conclusion

We here combined physiological synchrony and unsupervised learning techniques with the aim to identify groups of individuals sharing the same selective attentional focus. Clustering performance well above chance level was reached when using EEG, but above chance level accuracies were not reached when using EDA or heart rate alone. Obtained results differed depending on the used mapping and clustering algorithm, but applying mapping before clustering generally led to better results. Combining information from multiple modalities resulted in a higher classification performance in cases where EEG was combined with heart rate and/or EDA, and resulted in more robust performance across different types of mapping and clustering algorithms, making clustering results less dependent on the specific algorithm choice. These results may enable researchers to study attentional engagement in everyday settings. We suggest researchers to use a multimodal approach due to its robustness to specific algorithm choice, enabling more consistent and generally better clustering results.

## Data Availability Statement

Publicly available datasets were analyzed in this study. This data can be found here: https://osf.io/8kh36/.

## Ethics Statement

The studies involving human participants were reviewed and approved by TNO Institutional Review Board. The participants provided their written informed consent to participate in this study.

## Author Contributions

A-MB and IS conceived and designed the study. IS and NT performed the experiment. AM and IS performed data analysis with input from all authors and wrote the first drafts of the manuscript. All authors thoroughly reviewed and revised the manuscript and approved the submitted version.

## Funding

This work was supported by the Netherlands Organization for Scientific Research (NWA Startimpuls 400.17.602).

## Conflict of Interest

The authors declare that the research was conducted in the absence of any commercial or financial relationships that could be construed as a potential conflict of interest.

## Publisher's Note

All claims expressed in this article are solely those of the authors and do not necessarily represent those of their affiliated organizations, or those of the publisher, the editors and the reviewers. Any product that may be evaluated in this article, or claim that may be made by its manufacturer, is not guaranteed or endorsed by the publisher.
