# Supplementary material for: Unsupervised Clustering of Individuals Sharing Selective Attentional Focus Using Physiological Synchrony
Source: Front Neuroergon. 2022 Jan 4;2:750248. doi: 10.3389/fnrgo.2021.750248 (PMC10790845; doi:10.3389/fnrgo.2021.750248)
Supplement: Supplementary file 1 [file Table_1.pdf]

Table A1. Overview of classification results for all combinations of mapping algorithm and clustering algorithm for EEG, EDA and HR. Each cell presents – in that order – the classification accuracy, the silhouette coefficient and misclassified participant IDs.

Mapping	Clustering	EEG	EDA	HR
None	k-means	x	x	x
	Spectral	85%<br>0.154<br>[2, 3, 8, 18]	62%<br>-0.073<br>[0,1,3,5,8,9,10,19,20,25]	58%<br>0.013<br>[0,2,6,10,12,13,16,17,19,23,25]
	Hierarchical	65%<br>0.173<br>[0,2,3,7,8,10,16,18,25]	54%<br>0.207<br>[0,1,3,4,5,6,10,12,15,19,23,24]	58%<br>0.112<br>[0,2,6,10,11,12,13,16,17,23,25]
	k-medoids	No convergence	No convergence	No convergence
Spectral clustering map	k-means	77%<br>0.353<br>[2,3,8,10,16,18]	62%<br>0.418<br>[7,9,11,14,16,17,18,21,22,25]	65%<br>0.408<br>[0,2,5,7,11,15,18,19,25]
	Spectral	54%<br>0.341<br>[1,3,5,8,9,10,11,15,16,17,18,19]	54%<br>0.306<br>[1,7,8,9,11,14,16,17,19,21,22,24]	69%<br>0.279<br>[2,6,10,12,15,19,23,25]
	Hierarchical	65%<br>0.173<br>[0,2,3,7,8,10,16,18,25]	62%<br>0.418<br>[7,9,11,14,16,17,18,21,22,25]	58%<br>0.328<br>[2,6,9,10,12,13,16,17,19,23,25]
	k-medoids	x	x	x
PCoA	k-means	77%<br>0.409<br>[2,3,8,10,16,18]	62%<br>0.441<br>[7,9,11,14,16,17,18,21,22,25]	50%<br>0.352<br>*chance level*
	Spectral	85%<br>0.383<br>[2,3,8,18]	58%<br>0.311<br>[1,7,9,11,14,16,17,19,21,22,24]	58%<br>0.329<br>[0,2,6,10,12,13,16,17,19,24,25]
	Hierarchical	77%<br>0.409<br>[2,3,8,10,16,18]	62%<br>0.441<br>[7,9,11,14,16,17,18,21,22,25]	54%<br>0.329<br>[0,2,6,9,10,12,13,16,17,19,23,25]
	k-medoids	77%<br>0.409<br>[2,3,8,10,16,18]	62%<br>0.441<br>[7,9,11,14,16,17,18,21,22,25]	50%<br>0.352<br>*chance level*
mMDS (averaging)	k-means	73%<br>x<br>[2,3,8,10,16,18,25]	58%<br>x<br>[1,4,7,9,11,14,16,17,19,21,22]	62%<br>x<br>[2,6,10,12,13,16,17,19,23,25]
	Spectral	77%<br>x<br>[2,3,8,16,18,25]	58%<br>x<br>[1,4,7,9,11,14,16,17,19,21,22]	62%<br>x<br>[2,6,10,12,13,16,17,19,23,25]
	Hierarchical	73%<br>x<br>[2,3,8,10,16,18,25]	58%<br>x<br>[7,9,11,14,16,17,18,19,21,22,25]	69%<br>x<br>[0,2,7,10,11,15,19,25]
	k-medoids	73%<br>x<br>[2,3,8,10,16,18,25]	54%<br>x<br>[0,2,5,8,10,12,13,15,18,20,23,25]	62%<br>x<br>[2,6,10,12,13,16,17,19,23,25]

mMDS (PCoA initialization)	k-means	58%<br>0.318<br>[1,3,5,8,9,10,11,13,16,17,18]	50%<br>0.338<br>*chance level*	62%<br>0.334<br>[2,6,10,12,13,16,17,19,23,25]
	Spectral	73%<br>0.310<br>[2,3,8,10,11,18,25]	54%<br>0.337<br>[0,2,3,5,6,10,12,13,15,18,20,23,24,25]	62%<br>0.334<br>[2,6,10,12,13,16,17,19,23,25]
	Hierarchical	73%<br>0.312<br>[0,2,7,8,16,18,25]	62%<br>0.341<br>[7,9,11,14,16,17,18,21,22,25]	73%<br>0.309<br>[2,5,12,15,18,19,25]
	k-medoids	58%<br>0.318<br>[1,3,5,8,9,10,11,13,16,17,18]	62%<br>0.331<br>[0,5,10,12,13,15,18,20,23,25]	62%<br>0.334<br>[2,6,10,12,13,16,17,19,23,25]
nMDS (averaging)	k-means	69%<br>x<br>[0,2,3,8,10,16,18,25]	50%<br>x<br>*chance level*	58%<br>x<br>0,2,6,10,12,13,16,17,19,23,25
	Spectral	65%<br>x<br>0,2,3,8,10,11,16,18,25	54%<br>x<br>[1,4,7,9,11,14,16,17,19,21,22]	62%<br>x<br>[2,6,10,12,13,16,17,19,23,25]
	Hierarchical	77%<br>x<br>[2,3,8,16,18,25]	54%<br>x<br>[0,2,5,8,10,12,13,15,18,20,23,25]	62%<br>x<br>[2,6,10,12,13,16,17,19,23,25]
	k-medoids	65%<br>x<br>[0,2,3,8,10,11,16,18,25]	50%<br>0.338<br>*chance level*	58%<br>x<br>[0,2,6,10,12,13,16,17,19,23,25]
nMDS (PCoA initialization)	k-means	65%<br>0.339<br>[0,2,3,8,10,11,16,18,25]	65%<br>0.331<br>[0,10,12,13,15,18,20,23,25]	62%<br>0.331<br>[2,6,10,12,13,16,17,19,23,25]
	Spectral	x	58%<br>0.328<br>[1,4,7,9,11,14,16,17,19,21,22]	62%<br>0.331<br>[2,6,10,12,13,16,17,19,23,25]
	Hierarchical	54%<br>0.281<br>[1,3,5,8,9,10,11,15,16,17,18,19]	58%<br>0.282<br>[0,2,5,12,13,15,16,18,20,23,25]	73%<br>0.312<br>[2,5,12,15,18,19,25]
	k-medoids	62%<br>0.317<br>[2,3,5,8,9,10,11,16,17,18]	54%<br>0.314<br>[2,3,5,6,12,13,15,18,20,23,25]	62%<br>0.331<br>[2,6,10,12,13,16,17,19,23,25]
UMAP	k-means	65%<br>x<br>[0,2,3,8,10,11,16,18,25]	54%<br>x<br>[2,7,13,14,16,17,18,19,21,22,23,25]	62%<br>x<br>[1,3,5,7,8,11,15,21,22,25]
	Spectral	65%<br>x<br>[0,2,3,8,10,11,16,18,25]	58%<br>x<br>[0,1,3,4,5,6,7,9,11,22,24]	62%<br>x<br>[1,3,5,7,8,11,15,21,22,25]
	Hierarchical	65%<br>x	54%<br>x	62%<br>x

		[0,2,3,8,10,11,16,18,25]	[2,7,13,14,16,17,18,19,21,22,23,25]	[1,3,5,7,8,11,15,21,22,25]
	k-medoids	x	x	x
UMAP (self-supervised learning)	k-means	77%<br>x<br>[3,4,8,10,16,25]	54%<br>x<br>[0,2,3,7,8,10,11,14,15,19,21,25]	50%<br>x<br>*chance level*
	Spectral	77%<br>x<br>[3,4,8,10,16,25]	54%<br>x<br>[0,3,5,7,8,11,14,15,19,21,22,25]	54%<br>x<br>[0,3,7,8,10,11,14,15,19,21,22,25]
	Hierarchical	77%<br>x<br>[3,4,8,10,16,25]	58%<br>x<br>[0,3,7,8,10,11,14,15,19,21,25]	62%<br>x<br>[0,3,7,8,11,14,15,19,21,25]
	k-medoids	x	x	x

Table A2. Overview of classification results for all combinations of mapping algorithm and clustering algorithm for the multimodal combinations EEG - EDA, EEG – HR, EDA – HR and EEG – EDA – HR. Each cell presents – in that order – the classification accuracy, the silhouette coefficient and misclassified participant IDs.

Mapping	Clustering	EEG - EDA	EEG – HR	EDA – HR	EEG – EDA -HR
MVMDS	k-means	85%<br>0.295<br>[8,14,18,25]	92%<br>0.381<br>[2,19]	58%<br>0.371<br>[0,5,6,10,12,15,18,19,20,23,25]	81%<br>0.368<br>[2, 4, 7, 14, 24]
	Spectral	81%<br>0.287<br>[8,14,18,20,25]	88%<br>0.371<br>[2,7,19]	54%<br>0.310<br>[0,6,10,12,13,15,16,17,18,19,23,25]	85%<br>0.354<br>[2,7,14,24]
	Hierarchical	65%<br>0.358<br>[2,4,7,11,16,17,18,22,25]	85%<br>0.403<br>[2,14,19,24]	62%<br>0.355<br>[0,5,6,10,12,15,18,19,20,23]	69%<br>0.248<br>[0,12,13,16,17,18,23,25]
	k-medoids	x	x	x	x
MVMDS with rescaling	k-means	85%<br>0.321<br>[8,14,18,25]	92%<br>0.389<br>2,19	58%<br>0.378<br>[0, 5, 6, 10, 12, 15, 18, 19, 20, 23, 25]	88%<br>0.376<br>2,7,25
	Spectral	85%<br>0.321<br>[8,14,18,25]	88%<br>0.380<br>[2,7,19]	54%<br>0.323<br>[0,6,10,12,13,15,16,17,18,19,23,25]	92%<br>0.352<br>[14,25]
	Hierarchical	65%<br>0.369<br>[2,4,7,11,16,17,18,22,25]	84%<br>0.409<br>[2,14,19,24]	62%<br>0.358<br>[0,5,6,10,12,15,18,19,20,23]	92%<br>0.331<br>[14,24]
	k-medoids	x	x	x	x
MVSC	k-means	81%<br>0.614<br>[8,16,18,20,25]	85%<br>0.621<br>[2,12,19,24]	54%<br>0.652<br>[0,2,10,12,13,15,16,17,18,19,23,25]	85%<br>0.662<br>[2,7,16,25]
	Spectral	81%<br>0.614<br>[8,16,18,20,25]	85%<br>0.621<br>[2,12,19,24]	62%<br>0.615<br>[0,2,10,12,13,15,16,19,23,25]	85%<br>0.662<br>[2,7,16,25]
	Hierarchical	81%	81%	54%	85%

	0.614<br>[8,16,18,20,25]	0.620<br>[2,4,7,12,19]	0.652<br>[0,2,10,12,13,15,16,<br>17,18,19,23,25]	0.662<br>[2,7,16,25]
k-medoids	x	x	x	x
